# Hand-assisted living-donor nephrectomy: a retrospective comparison of two techniques

**DOI:** 10.1186/s12894-018-0355-2

**Published:** 2018-05-10

**Authors:** Jeannette D. Widmer, Andrea Schlegel, Philipp Kron, Marc Schiesser, Jens G. Brockmann, Markus K. Muller

**Affiliations:** 1 Division of Visceral and Transplantation Surgery, University Hospital, Zurich, Switzerland; 2 Department of Surgery, Kantonsspital Frauenfeld, 8500 Frauenfeld, Switzerland; 3 Department of Surgery, Kantonsspital St. Gallen, St. Gallen, Switzerland; 4 Department of Surgery, Kidney and Pancreas Transplantation, King Faisal Specialist Hospital, Riyadh, Kingdom of Saudi Arabia

## Abstract

**Background:**

Living-donor nephrectomy (LDN) is challenging, as surgery is performed on healthy individuals. Minimally invasive techniques for LDN have become standard in most centers. Nevertheless, numerous techniques have been described with no consensus on which is the superior approach. Both hand-assisted retroperitoneoscopic (HARS) and hand-assisted laparoscopic (HALS) LDNs are performed at Zurich University Hospital. The aim of this study was to compare these two surgical techniques in terms of donor outcome and graft function.

**Method:**

Retrospective single-center analysis of 60 consecutive LDNs (HARS *n* = 30; HALS *n* = 30) from June 2010 to May 2012, including a one-year follow-up of the recipients.

**Results:**

There was no mortality in either group and little difference in the overall complication rates. Median warm ischemia time (WIT) was significantly shorter in the HARS group. The use of laxatives and the incidence of postoperative vomiting were significantly greater in the HALS group. There was no difference between right- and left-sided nephrectomies in terms of donor outcome and graft function.

**Conclusions:**

Both techniques appear safe for both donors and donated organs. The HARS technique is associated with a shorter WIT and a reduced incidence of postoperative paralytic ileus. Therefore, we consider HARS LDN a valuable alternative to HALS LDN.

## Background

For patients with end-stage renal disease, kidney transplantation is the only treatment to improve their survival, and it quickly enhances their quality of life [1, 2]. During the last several decades, living-donors have become the primary source of donor kidneys for transplantation [3]. As well as expanding the donor organ pool, living-donor kidneys allow preemptive transplantation, and surgery can be efficiently planned with both the donor and the recipient in optimum medical condition. Hence, the outcome of living-donor kidney transplantation is superior to transplantation of deceased donor kidneys in terms of improved long-term recipient survival, quality of life, early graft function and better graft survival [4–6]. However, living-donor nephrectomy (LDN) remains a surgical challenge in terms of minimizing postoperative complications as it is performed on healthy individuals. Therefore, the most important goal is ensuring the safety and well-being of the voluntary donor [7–9]. The surgical technique must result in the least possible risk of morbidity without compromising the functional outcome of grafts.

The first laparoscopic donor nephrectomy was performed in 1995 by Kavoussi and Ratner [10] and became a well-established technique due to many advantages, including less postoperative pain, a shorter hospital stay and better cosmetics, when compared to open surgical approaches [11, 12]. In contrast, the prolonged warm ischemia time (WIT) has been highlighted as the major disadvantage [13, 14]. A compromise was found in the hand-assisted laparoscopic (HALS) donor nephrectomy technique, which was first described by Wolf et al. in 1998 [15]. This approach shortens the WIT compared to a purely laparoscopic nephrectomy. Further advantages include tactile feedback, easier and rapid control of bleeding by digital pressure and more rapid kidney recovery [16, 17]. Injuries to the bowel and/or other organs are more likely by a transperitoneal approach due to its nature of abdominal access [18]. In 2001, Wadström et al. combined the advantages of HALS nephrectomy and the retroperitoneoscopic approach favored by urologists and introduced the hand-assisted retroperitoneoscopic (HARS) LDN technique [19]. Direct access to the kidneys without entering the peritoneal cavity obviates bowel injury and is associated with shorter operating time, shorter WIT, less blood loss and a shorter length of hospital stay compared to HALS LDN [20, 21].

The aim of this study was to compare these two surgical techniques in terms of donor outcome and graft function.

## Methods

### Donors

All donors undergoing HARS or HALS LDNs at the University Hospital of Zurich between June 2010 and May 2012 and the corresponding recipients were identified. A preoperative work-up was carried out according to our standard donor protocol (conformable to the Amsterdam forum guidelines [22]). Indications and results were discussed by the donor kidney board, which is a panel of transplant surgeons, nephrologists, transplantation coordinators, and immunology and psychology experts who meet on a weekly basis. The decision of which donor kidney to retrieve was based first on functional investigations. In the case of functional differences of less than 3%, anatomical considerations guided surgical decision-making. The guiding principle was to leave the better, greater-functioning organ with the donor. The choice of surgical technique depended on the preference of the lead surgeon. All donors received standard postoperative analgesia plus on-demand medication. Donors were followed-up with in our surgical outpatient clinic at 3 months after surgery.

### Surgical procedure

For both techniques, the donor was placed in the contralateral flank position with slight hip flexion, thereby extending the ipsilateral flank.

HARS technique: A Pfannenstiel incision was made to introduce the hand-port (GelPort™, Applied Medical Resources Corporation). The retroperitoneal space was created by blunt dissection. The introduction of the first trocar (camera) in the lateral upper abdomen was guided by the surgeon’s hand, followed by insufflation of carbon dioxide up to 12 cm H_2_O. One or two additional working trocars were placed under direct vision in the medial lower abdomen. Identification and dissection of the kidney, ureter and vascular structures were performed by hand and with an endoscopic device (Harmonic Ultrasonic Shears, Ethicon). Following complete dissection of all structures, the ureter was clipped and transected distally, and the renal vein and artery were accurately exposed and then divided by a stapler device. The kidney was recovered through the open hand-port.

HALS technique: A periumbilical camera port was introduced into the abdominal cavity, followed by insufflation of carbon dioxide up to 15 cm H_2_O. Two or three additional working trocars were placed in the lateral upper and lower abdomen under direct vision. Before clipping the ureter and dividing the renal vein and artery, the hand-port was introduced through a Pfannenstiel incision. After full mobilization, the kidney was extracted through the open hand-port.

Abdominal closure for both techniques was performed in a standard fashion. Drains were not routinely placed.

### Recipients

All renal grafts were placed extraperitoneally into the iliac fossa. The immunosuppressive regimen was determined preoperatively depending on immunological and donor-specific risk stratification. Postoperative care was undertaken by the surgical lead. Recipients were followed-up regularly with by the Department of Nephrology at the University Hospital of Zurich.

### Data collection

Demographic and clinical data were retrospectively obtained from the clinical information system, which maintains all patients’ data electronically. The following parameters from donors and recipients were retrieved: preoperative renal retention parameters, BMI, blood group, and HLA status. Operative time (OT), WIT and organ site were noted. Kidney function (creatinine, GFR) before and after surgery were recorded for both donors and recipients. Delayed graft function (DGF) was defined as the use of dialysis in the first postoperative week [23]. In terms of postoperative parameters, the time to first bowel movement, laxative use, postoperative vomiting, use of analgesics and length of hospital stay were recorded. Complications were routinely ranked using the Clavien-Dindo classification, a five-grade therapy-based system in which higher grades reflect more severe complications [24]. In this study, the Comprehensive Complication Index (CCI), a new scale to better quantify postoperative morbidity, was also applied [25]. All complications arising from a single case and ranked by the Clavien-Dindo classification were summarized and expressed on a scale of 0 to 100. The calculation was made using the CCI® calculator (www.assessurgery.com). The CCI score allows quantification of all complications after a surgical procedure, while the Clavien-Dindo classification describes only the most severe complication. Donors were followed-up until 90 days after surgery.

### Statistical analysis

Data analysis was performed using SPSS 21 (SPSS Inc., Chicago IL, USA). All data values presented are median values with an interquartile range (IQR) unless otherwise stated. A bivariate analysis was carried out comparing selected variables in the HALS and HARS cohorts. Differences in continuous variables between groups were tested for statistical significance using the Mann-Whitney U test. For comparison of proportions Fisher’s exact tests was used. *P* values < 0.05 were considered statistically significant.

### Availability of data and materials

The datasets used and/or analyzed during the current study are available from the corresponding author on reasonable request.

## Results

### Donor

#### Demographic and clinical data

A total of 60 live kidney donors were included in the study (*n* = 30 HARS, *n* = 30 HALS). The demographic and clinical characteristics of the kidney donors are summarized in Table 1. There was no relevant difference between the two groups.

**Table 1:** Demographic and clinical characteristics of kidney donors

	HARS (*n* = 30)	HALS (*n* = 30)	*p*-value
Age (years)	56 (17)	50 (18)	0.268
Female	21 (70)	19 (63.3)	0.784
BMI (kg/m^2^)	24.8 (4.4)	25.5 (3.8)	0.765
Art. hypertension	4 (13.3)	5 (16.6)	1.000
Right-sided donation	16 (53.3)	12 (40)	0.437

Values are median and IQR, frequencies are in absolute numbers and %

### Perioperative data

The median OT did not differ considerably between groups (Table 2). Conversion to laparotomy was never required. In the HARS group, 16 (53.3%) patients underwent right-sided donor nephrectomy, compared to 12 (40%) in the HALS group. There was no relevant difference between right- and left-sided nephrectomies within the two groups with regard to postoperative renal function. There was a trend for the OT to be shorter in both groups for right-sided nephrectomies, but these differences did not reach statistical significance (HARS: 132 min versus 145 min, *p* = 0.15; HALS: 140 min versus 180 min, *p* = 0.06).

**Table 2:** Donors’ intra- and postoperative data

	HARS (*n* = 30)	HALS (*n* = 30)	*p*-value
OT overall (min)	140 (34)	170 (47)	0.060
- OT right-sided (min)	132 (40)	140 (52)	0.315
- OT left-sided (min)	145 (75)	180 (28)	0.197
WIT (sec)	120 (49)	150 (16)	0.008
- WIT of right kidneys (sec)	120 (38)	150 (23)	0.208
- WIT of left kidneys (sec)	120 (60)	150 (4)	0.017
First bowel movement (d)	2.1 (0)	2.4 (1)	0.385
Use of laxatives	19 (63.3)	27 (90)	0.030
Vomiting ^a^	4 (13.3)	7 (23.3)	0.506
- plus laxatives	1/4	7/7	0.010
Length of hospital stay (d)	6 (3)	6 (3)	0.062
Creatinine (umol/l)			
- Preoperative	69 (16)	69 (14)	0.646
- Postoperative	99 (28)	102 (26)	0.794
- Follow-up	104 (23)	100 (21)	0.556

*OT* operative time, *WIT* warm ischemia time

Values are median and IQR, frequencies are in absolute numbers and % ^a^vomiting after 24 h and 7 days postoperatively

Median WIT was significantly shorter in the HARS group than in the HALS group (120 s versus 150 s, *p* = 0.008). There was no relevant difference in WIT for left-sided compared to right-sided organ harvest for both surgical techniques.

The demand for postoperative analgesia was similar between groups. Median time to first bowel movement after surgery did not differ between the HARS and HALS groups. However, there was a significant difference in the use of laxatives: 19 (63.3%) donors in the HARS group received laxatives compared to 27 (90%) in the HALS group. In the HARS group, 4 (13.3%) patients experienced at least one episode of postoperative emesis (24 h - 7 days) compared to 7 (23.3%) in the HALS group. All HALS donors experiencing emesis required laxatives, while this was the case for only one donor in the HARS group.

It is institutional policy that donors can decide how long they would like to stay in the hospital following surgery, and their wishes are accommodated. However, the median length of hospital stay did not differ between the two groups. The median creatinine preoperatively, before discharge, and during 3 months of follow-up did not differ between the two groups.

### Minor complications (grade 0-II)

Postoperative courses were uneventful in 22 (73.3%) donors in the HARS group and 18 (60%) in the HALS group (Table 3).

**Table 3:** Donor complications according to the Clavien-Dindo score and CCI within 90 days

	HARS (*n* = 30)	HALS (*n* = 30)	*p*-value
Clavien-Dindo Score			
Grade I	2 (6.6)	2 (6.6)	0.672
Grade II	3 (10)	6 (20)	
Grade IIIb	3 (10)	4 (13.3)	
None	22 (73.3)	18 (60)	
CCI			
Overall	6.16 (11.26)	10.36 (15.28)	0.245
Right-sided LDN	6.83 (12.25)	8.18 (11.97)	0.775
Left-sided LDN	5.39 (9.96)	11.70 (16.98)	0.307

Values are mean and SD, frequencies are in absolute numbers and %

Two (6.6%) Grade I complications occurred in each group, including incisional pain, lumbago and shoulder ache. They all could be eased by either subcutaneous infiltration of local anesthetics or oral analgesia and physiotherapy. All Grade I complications corresponded to a CCI of 8.7 points.

Three (10%) Grade II complications occurred in the HARS group, including seroma, hematoma and a new onset of arterial hypertension. All complications required medical treatment with antibiotics combined with frequent wound hygiene or antihypertensive drugs and corresponded to a CCI of 20.9 points. Within the HALS group, six (20%) Grade II complications were observed, including newly diagnosed arterial hypertension, fever of unknown origin and wound seroma. All complications could be treated by drug therapy and corresponded to a CCI of 20.9 points except a donor with two postoperative complications (Grade I and Grade II), whose CCI totaled 22.6 points.

### Major complications (grade III-V)

Three donors in the HARS group underwent surgical evacuation of subcutaneous hematoma in the area of the Pfannenstiel incision (Grade IIIb). The CCI was 33.7 points in all three cases. Four Grade IIIb complications occurred in the HALS group. Two subcutaneous hematomas required operative evacuation. One subcutaneous infection had to be treated surgically, followed by vacuum-assisted wound closure. One of those donors additionally underwent subcutaneous infiltration of local anesthetic (Grade I) due to persistent incisional pain. In that donor, the CCI was 34.8 points. The others corresponded to a CCI of 33.7 points. In addition, one donor underwent three surgical revisions (two subcutaneous evacuations of hematoma and one exploratory laparotomy) for a severe hemorrhage. During the exploratory laparotomy, laceration of the spleen and the small bowel mesentery was found. Treatment consisted of a splenectomy and mesenteric suture. The same donor developed a hypertensive crisis during postoperative monitoring in the ICU that required intravenous antihypertensive medication. The sum of complications in this donor resulted in a CCI of 58.4 points. High-grade complications, i.e., single- or multi-organ failure (Grade IVa or IVb) as well as donor death (Grade V) did not occur in either group.

### Recipients

#### Demographic and clinical data

The recipients’ detailed preoperative characteristics are shown in Table 4. In the HARS group, 20 (66%) recipients underwent dialysis for a median of 8 months prior to transplantation; 14 had hemodialysis and 6 had peritoneal dialysis. In the HALS group, 23 (77%) recipients were dialyzed preoperatively for a median of 10 months, with 22 by hemodialysis and 1 by peritoneal dialysis.

**Table 4:** Demographic and clinical characteristics of recipients’ data

	HARS (*n* = 30)	HALS (*n* = 30)	*p*-value
BMI (kg/m^2^)	23.9 (5.8)	25.6 (5.4)	0.174
Preoperative Dialysis	20 (3.3)	23 (76.6)	0.567
- Hemodialysis	14 (46.6)	22 (73.3)	
- Peritoneal Dialysis	6 (20)	1 (3.3)	
First Kidney-Tx	24 (80)	29 (96.6)	0.103
Retransplantation	6 (20)	1 (3.3)	0.103
AB0-Incompatibility	5 (16.6)	5(16.6)	1.000
HLA			
- 0-2 mismatches	3	5	0.819
- 3-4 mismatches	12	15	0.604
- 5-6 mismatches	15	10	0.294
OT (min)	106 (39)	120 (20)	0.271
DGF/PNF	4	1	0.353
- PNF	1	0	
Creatinine (umol/l)			
- Before discharge	110 (47)	108 (41)	0.996
- After one year	118 (31)	115 (37)	0.605

Values are median and IQR, frequencies are in absolute numbers and %, *Tx* Transplantation, *DGF* delayed graft function, *PNF* primary non-function

### Perioperative data

There were no relevant differences between the two groups in the median serum creatinine level and creatinine clearance before discharge and at 1 year after transplantation.

### Survival and graft function

The recipients’ one-year survival rate was 93.3% in the HARS group and 100% in the HALS group. The one-year graft survival rate was also comparable in both groups (90% versus 93.3%). In the HARS group, 29 recipients (96.6%) had an immediate or delayed onset of diuresis compared to all recipients (100%) in the HALS group (Table 4).

The patient with primary non-function in the HARS group developed multiple complications, starting with an acute vascular rejection from the very beginning. In the further course a dehydrating diarrhea due to a pseudomembranous colitis occurred, followed by intestinal bleeding necessitating a hemicolectomy, and finally sepsis and multiple organ failure. The patient died 14 weeks after transplantation.

There were three cases of DGF in the HARS group and one in the HALS group. One of the DGF patients in the HARS group developed a subcutaneous hematoma postoperatively, which led to a hemodynamic instability and required two units of blood prior to the occurrence of diuresis. The second patient with DGF had a perforation of the cecum due to ischemic colitis, necessitating an ileocecal resection. The patient developed a burst abdomen postoperatively because of an abscess in the renal graft bed. The kidney, which worked perfectly before the ileoceacal resection, did not recover from this septic burst and was explanted 4 months after transplantation. The third recipient suffered from a very severe diarrhea postoperatively resulting in a metabolic derailment, which led to a temporary loss of kidney function. This recipient later developed necrotizing pancreatitis, resulting in permanent loss of graft function and the development of multiple organ failure 6 weeks after transplantation, and died. The DGF patient in the HALS group underwent immediate surgical revision due to bleeding from the venous anastomosis. Temporary hemodialysis was required, and the kidney did partially recover; the GFR was 21 ml/min at the one-year follow-up, but dialysis was not yet needed.

## Discussion

There are two different approaches for hand-assisted donor nephrectomy: transabdominal and retroperitoneal. Evidence of the superiority of either technique is lacking. This analysis has shown equivalent donor outcomes after both techniques in terms of postoperative kidney function and complications. The overall WIT was significantly shorter for kidneys recovered by the retroperitoneal approach despite similar operating times for the two surgical techniques. There does, however, seem to be a trend for a shorter OT using the HARS approach. Laxatives were used significantly more often in the HALS group, and concurrent vomitus was observed in all of these patients, suggesting that the transabdominal technique has a greater effect on bowel function. Finally, both left and right nephrectomies appear to be safe and feasible with both surgical techniques.

The first LDN was performed in 1954 with an open approach [26], which remained the gold standard for more than 40 years. In 1995, a purely laparoscopic technique to procure kidneys from living-donors was pioneered [10]. Wolf et al. [15] described the HALS donor nephrectomy in 1998 for the first time, followed by Wadström et al. [19], who introduced the HARS nephrectomy 3 years later. Various advantages soon became obvious, such as the ability to use tactile feedback, easier and rapid control of bleeding by digital pressure, better exposure and dissection of structures, and more rapid kidney recovery [16]. Studies of HALS or HARS procedures describe superiority compared to purely laparoscopic nephrectomies with regard to OT and WIT [18]. Therefore, the laparoscopic approach with or without hand-assistance has become the gold standard at most kidney transplant centers [27].

Postoperative donor complication rates range from 0 to more than 40% in different reports, depending on how authors classify adverse events after a nephrectomy [20, 28–30]. Our analysis defined complications according to the Clavien-Dindo classification as well as to the CCI [24, 25]. The latter is a new scale to measure surgical morbidity and is not yet routinely used in clinical practice. Therefore, we were interested in its applicability to living kidney donors as well as in how it compares to the Clavien-Dindo classification. First, there were no mortalities or life-threatening complications except for one donor who required an emergency relaparotomy and splenectomy. There were no conversions from laparoscopic to open surgery. We found no relevant differences in graft survival and function between the two groups at the one-year follow-up. DGF occurred in three (10%) recipients transplanted with a kidney recovered by the retroperitoneal approach and in one (3.3%) patient with a kidney harvested by the transperitoneal approach. DGF as well as PNF (primary non-function, defined as permanent loss of allograft function starting immediately after transplantation) certainly are very important indicators for graft function and should not be neglected. In this regard the definition of DGF is crucial. We defined DGF as needed dialysis within the first week after transplantation [23]. In the literature, the frequency of DGF varies from 0 to 10% in living-donor transplantation, indicating that the presented results are within the expected range [28, 31, 32]. We hypothesized that the graft functions were delayed or not existing due to complications in the recipient. Either there was a hemodynamic instability postoperatively due to a subcutaneous bleeding, a sepsis because of a cecal perforation during the first week after transplant surgery, a severe diarrhea postoperatively which led to a metabolic derailment followed by a secondary anuria, an acute vascular rejection or a bleeding from the venous anastomosis. But still, contributing factors during the donor kidney harvesting cannot be totally ruled out. The overall donor complication rate according to the Clavien-Dindo classification during follow-up tended to be higher, though not significantly, in the HALS group for in-hospital minor adverse events. However, Dols et al. [21] reported in a randomized controlled trial an increased incidence for high-grade complications in HALS compared to HARS nephrectomy patients. On the other hand, Ruszat et al. [20] documented overall complication rates of 20% for the retroperitoneoscopic and 15% for the hand-assisted transabdominal approach. Interestingly, these complication rates were significantly lower than those for purely laparoscopic nephrectomies (42.9%). Analysis of the CCI of our donors showed higher scores in the HALS group compared to the HARS group, although these differences did not reach statistical significance. The CCI better represents the severity of adverse events than does the Clavien-Dindo Score. In our opinion, the new CCI scoring system is applicable and yields more information about overall complications following donor nephrectomy.

There was no relevant difference in the OT between the two techniques in our analysis, although there was a trend for a longer OT in the HALS group, mirroring other published results [21]. A key difference between the two techniques that may affect the OT is the timing of when the surgeon installs the hand-port and inserts his/her hand. In the HARS technique, hand assistance is part of the procedure from the very beginning, while in the HALS technique, hand assistance is only used for dissecting the vascular structures and for recovery of grafts. Whether this influences the recorded time of the operation remains speculative. OT is an important consideration, as there is sufficient evidence that both renal function and organ perfusion are compromised by the duration of pneumoperitoneum [33, 34]. The WIT was significantly shorter in our study for kidneys that were recovered by the retroperitoneal approach. Given the various definitions of WIT that exist, it is difficult to compare WITs across different studies. It has been previously reported that the WIT in the HARS technique is shorter than in the HALS technique [20, 35]. The differences in the WIT measured in our study did not translate into differences in delayed graft function, graft loss or renal function. This is consistent with previously published results [31].

Our study indicates for the first time that patients undergoing HALS procedures require significantly more laxatives compared to those undergoing HARS procedures. All of the donors in the HALS group who vomited after 24 h postoperatively required laxatives in contrast to only 25% in the HARS group. This is perhaps not surprising, but it confirms the hypothesis that patients undergoing transperitoneal nephrectomies are more frequently afflicted with a paralytic ileus. Still, other factors (e.g. OT) may have influenced these results. Matas et al. compared morbidity rates among open, hand-assisted laparoscopic and pure laparoscopic nephrectomies [7]. Bowel obstruction also occurred significantly more often in the hand-assisted laparoscopic group then in the others. Other studies showed more serosa and splenic lesions in transperitoneal approaches [18].

There is repeated discussion concerning the preference for right or left donor nephrectomies. Most centers favor left kidneys for donation because of the longer renal vein, which makes implantation less demanding. On the other hand, some surgeons prefer the right kidney because it is easier to retrieve. A recent Meta-Analysis demonstrate a significant difference in terms of OT in favor of right-sided HALS nephrectomies [36]. In our study, there was a trend for a shorter OT in both groups for right-sided nephrectomies, although these differences did not reach statistical significance. A shorter OT for the right kidney can be expected due to the lower position of the right kidney and generally no adrenal, lumbar or genital veins draining into the right renal vein. There is also less need for colonic dissection and no risk of lacerating the spleen. Our OTs for right- and left-sided nephrectomies in both groups are shorter overall when compared to results of other similar studies [16, 37]. Eventually, the OT depends on the experience of the performing surgeon. The donor complication rates were comparable and did not show a relevant difference between right- and left-sided kidney donations.

An obvious limitation of our study is the small number of patients and, therefore, the low statistical power. The retrospective nature of the study is also a limitation. Only two surgeons carried out the donor procedures and patients were not randomized for the type of surgery performed. Hence, the procedures were always performed in the same way, and because both consultant surgeons were experienced, the results did not vary over time. The fact that the hand assistance in the two groups was at different time points during the procedure limits the direct comparison of the two groups. Recipient surgery in most of the cases was performed by just one of the two surgeons. Therefore, recipient surgery does not bias the presented results.

With respect to donor safety, surgeons should apply the surgical technique they are best trained in and feel most comfortable with. Future training of new surgeons should include various techniques to give them the confidence to use the best surgical approach to match individual circumstances and to be versatile enough to change procedures if research determines the superiority of one procedure. Furthermore, long-term follow-up is urgently needed because information about outcome (e.g., late complications caused by abdominal adhesions) over a longer time period in both groups is scarce.

## Conclusion

There were no mortalities or intraoperative challenges leading to conversion in either donor group. In agreement with the current literature, it was demonstrated that HARS and HALS approaches for LDN are safe and feasible for renal graft recovery on either side. Neither of the two techniques provided clear evidence of superiority. And we found no relevant difference in graft function after 1 year postoperatively. However, given that the WIT is significantly shorter and the incidence of postoperative paralytic ileus is less frequent in the HARS group, we suggest that this technique might be preferred for living related kidney organ donors. Further advantages might be found for more complex patients, such as obese donors or donors with previous abdominal surgery.

## Abbreviations

BMI: Body mass index
CCI: Comprehensive complication index
DGF: Delayed graft function
GFR: Glomerular filtration rate
HALS: Hand-assisted laparoscopic
HARS: Hand-assisted retroperitoneoscopic
LDN: Living-donor nephrectomy
OT: Operative time
WIT: Warm ischemia time
